# Performance of the Sardinia Radio Telescope Using the Dual-Polarized Cryogenic C-Low Receiver in the 4.2–5.6 GHz Frequency Band

**DOI:** 10.3390/s26020698

**Published:** 2026-01-21

**Authors:** Luca Schirru, Elise Egron, Adelaide Ladu, Francesco Gaudiomonte, Alessandro Attoli, Alessandro Cabras, Giuseppe Carboni, Francesca Loi, Paolo Marchegiani, Marco Marongiu, Sara Mulas, Matteo Murgia, Mauro Pili, Alberto Pellizzoni, Sergio Poppi, Fabio Schirru, Valentina Vacca

**Affiliations:** National Institute for Astrophysics (INAF), Cagliari Astronomical Observatory, 09047 Selargius, Italy; elise.egron@inaf.it (E.E.); adelaide.ladu@inaf.it (A.L.); francesco.gaudiomonte@inaf.it (F.G.); alessandro.attoli@inaf.it (A.A.); alessandro.cabras@inaf.it (A.C.); giuseppe.carboni@inaf.it (G.C.); francesca.loi@inaf.it (F.L.); paolo.marchegiani@inaf.it (P.M.); marco.marongiu@inaf.it (M.M.); sara.mulas@inaf.it (S.M.); matteo.murgia@inaf.it (M.M.); mauro.pili@inaf.it (M.P.); alberto.pellizzoni@inaf.it (A.P.); sergio.poppi@inaf.it (S.P.); fabio.schirru@inaf.it (F.S.); valentina.vacca@inaf.it (V.V.)

**Keywords:** Sardinia Radio Telescope (SRT), C-band, cryogenic radio receiver, radio astronomy, radio frequency interference (RFI)

## Abstract

The Sardinia Radio Telescope (SRT) is an Italian antenna utilized for scientific research in the field of radio astronomy across a broad frequency range from 300 MHz to 116 GHz. Among the various cryogenic receivers installed on SRT, the dual-polarized C-Low receiver operates within the frequency range of 4.2–5.6 GHz, which is the lower portion of the well-known C-band, and is installed at the Gregorian focus of the telescope. This article presents a general description of the design of the receiver, highlighting its signal acquisition chain, which conditions weak signals from the sky for transmission to the digital back-end, responsible for data processing. An analysis of the radio-frequency interference environment affecting scientific observations is also presented, together with the adopted mitigation strategies. Finally, we report the results of the characterization tests performed with the C-Low receiver at SRT, focusing on the pointing accuracy model, gain-curve calibration, focus-curve calibration, and beam-shape analysis. The results of these characterization tests demonstrate the performance and accuracy of the C-Low receiver, providing a reference for future observations and instrumentation improvements.

## 1. Introduction

Radio astronomy is a branch of astronomy that studies celestial objects by observing the radio waves they emit. Unlike traditional optical astronomy, which relies on visible light, radio astronomy uses specialized instruments called radio telescopes to detect and analyze radio frequency signals from space. These signals, generally weak, can originate from a wide variety of sources, including stars, galaxies, pulsars, quasars, and the cosmic microwave background radiation [1]. A major advantage of radio waves is their ability to penetrate dust clouds that block visible light, enabling radio astronomers to observe regions of space that would otherwise be hidden, such as the centers of galaxies or star-forming areas [2,3]. Radio waves emitted by astronomical sources propagate across a wide range of frequencies. This article focuses on the C-band, which spans from 4 GHz to 8 GHz [4], with particular emphasis on the frequency window between 4.2 GHz and 5.6 GHz, also known as the lower part of the C-band. It includes important spectral lines like the formaldehyde mega maser emission at 4.83 GHz, useful for studying star-forming regions [5]. Additionally, this frequency range is particularly useful for observing continuum emission from galaxies, active galactic nuclei (AGN) [6], and pulsars [7]. The band is also employed for studies of neutral hydrogen (HI) absorption and molecular transitions [8].

Unfortunately, the 4.2–5.6 GHz band is occupied by many civilian services like radar, communication satellites, terrestrial sources and wireless transmission, which generate radio frequency interference (RFI) that impacts radio astronomy observations [9,10,11,12]. Furthermore, a considerable amount of unwanted signals can be produced by the numerous electronic devices commonly used in radio telescope control systems, posing a potential threat to the observational frequency bands. According to the International Telecommunication Union Radio Regulations (ITU-RR), the Radio Astronomy Service (RAS) plays a crucial role in managing frequency allocations dedicated to radio astronomy [13]. It provides protection for specific frequency bands, such as the 4.8–5 GHz window within the C-band, ensuring they can be used for scientific research without interference from other radio services [13]. This regulatory framework is crucial for maintaining the quality and accuracy of astronomical observations. Additionally, periodic RFI measurement campaigns are essential for gaining a comprehensive understanding of the RFI environment surrounding a telescope [14,15,16,17,18].

Around the world, several radio telescopes operate within this frequency range, and more broadly in the C-band. For example, the 100-m Effelsberg Radio Telescope [19], the 64-m Commonwealth Scientific and Industrial Research Organization (CSIRO) Parkes Radio Telescope (also known as “The Dish”) [20], and the 100-m Robert C. Byrd Green Bank Telescope (GBT) are all equipped with cryogenic receivers designed to work within this range of frequencies [21].

In the context of the Italian scene, the 64-m Sardinia Radio Telescope (SRT) is a cutting-edge Gregorian antenna owned by the Italian National Institute for Astrophysics (INAF) and managed by the Cagliari Astronomical Observatory (OAC) [22,23]. Situated in the Pranu Sanguini area, approximately 35 km northeast of Cagliari (Latitude 39.493072° N, Longitude 9.245151° E), SRT stands at an elevation of around 650 m above sea level. Designed to cover a broad frequency range from 300 MHz to 116 GHz, the telescope is equipped with several cryogenic receivers, including the historic L-P band receiver (1.3–1.8 GHz and 0.25–0.46 GHz) [14,24], the C-high receiver (5.7–7.7 GHz) [25], and the multi-feed K-band receiver (18–26.5 GHz) [26]. Recent additions, made possible by funding from the National Operational Program (PON) of the Italian Ministry of University and Research [27], include advanced receivers such as the multi-feed Q-band receiver (33–50 GHz) [28], the multi-feed W-band Cryogenic Array Receiver for Users of the Sardinia Observatory (CARUSO) receiver (70–116 GHz) [29], the tri-band receiver (K-band at 18–26 GHz, Q-band at 34–50 GHz, and W-band at 80–116 GHz) [30], and the W-band Milli-metric Sardinia radio Telescope Receiver based on Array of Lumped elements kinetic inductance detectors, known as MISTRAL (77–103 GHz) [31]. All these receivers are currently under technical and scientific commissioning. As part of the scheduled shutdown of the SRT required for the installation of the PON project receivers, a new receiver called the C-low receiver was also added in October 2023. Its name derives from the fact that it operates within the lower window of the C-band, covering frequencies from 4.2 to 5.6 GHz [28]. All receivers are installed at the focal points of SRT, which has six focal positions thanks to its multi-reflector design, with some positions (i.e., the primary focus and the Gregorian focus) able to host multiple receivers via dedicated mechanical structures. For further details about the design of SRT, including its optics and technical specifications, please refer to [22,23]. One of the standout technological features of SRT is its active surface, which compensates for gravitational deformation, thermal gradients, and wind pressure affecting the 64-m dish [32]. This is achieved through an electro-mechanical control system, ensuring high antenna efficiency across medium to high frequencies. SRT is equipped with several digital back-ends, useful for specific applications with each receiver, such as the Total Power (TP), Digital Filter Bank mark 3 (DFB3), Digital Base Band Converter (DBBC), Sardinia roach2-based digital architecture for radio astronomy (SARDARA) and Square Kilometer Array Reconfigurable Application Board (SKARAB) [33,34,35].

Periodic characterization tests are carried out at the SRT to evaluate the antenna’s condition, verify the proper functioning of its components, such as the active surface, receivers, and back-ends, and improve observing performance at various frequencies. These tests specifically include measurements of pointing accuracy, focus, gain curves, and beam shape, for the different receivers. Additionally, RFI measurement campaigns are periodically performed in order to update the RFI scenario around the telescope site [13,14,15,16,17,18].

This article presents the aforementioned C-low receiver of the SRT and reports the results of characterization tests performed on pointing accuracy, focus curve calibration, gain curves calibration, and beam shape characterization during the second half of 2024 and January 2025. These results provide a comprehensive assessment of the receiver’s performance within the 4.2–5.6 GHz frequency range, validating its operational reliability. On the basis of these verified characteristics, the C-low receiver has been instrumental from the outset for both very long baseline interferometry (VLBI) observations [36] and a variety of ongoing scientific projects, whose data are still under analysis.

Section 2 provides a detailed overview of the receiver’s design, along with an analysis of the unwanted signals detected within the frequency band of the receiver. Section 3 presents the results of the characterization tests, including the pointing accuracy model (Section 3.1), focus curve calibration (Section 3.2), gain curve calibration (Section 3.3), and beam shape characterization (Section 3.4). Finally, Section 4 concludes the study and outlines potential directions for future development.

## 2. Materials and Methods: The Dual-Polarized Cryogenic C-Low Receiver of the Sardinia Radio Telescope and the Scenario of Radio Frequency Interference

The dual-polarized cryogenic C-low receiver is installed on the Gregorian receiver positioner, a rotating octagonal turret mounted eccentrically on the Gregorian focal plane of SRT and designed to accommodate up to eight different receivers. Although the Gregorian focus of SRT is optimized to guarantee high performance in the 7.5–116 GHz frequency range, the C-low receiver (operating at 4.2–5.6 GHz) was nonetheless installed at this focal position. Originally, the receiver was designed to be mounted at the Beam WaveGuide (BWG) focus of SRT [22,23,28]. However, practical constraints—most notably the absence of the fourth mirror M4 required to redirect the radiation along the BWG optical path—led to a change of approach. For this reason, the receiver was reallocated to the Gregorian focus, and the feed design was subsequently optimized to ensure proper illumination and performance in this configuration [28]. A detailed description of the optical system of SRT can be found in references [22,23].

The design of the C-low receiver, from the feed horn to the digital back-end, is described in Section 2.1. Additionally, Section 2.2 covers some considerations regarding the RFI environment around the telescope, including unwanted signals that the SRT can detect with the C-low receiver from the surrounding territory.

### 2.1. Design of the C-Low Receiver and the Signal Acquisition Chain of the Sardinia Radio Telescope

**Figure 1:**
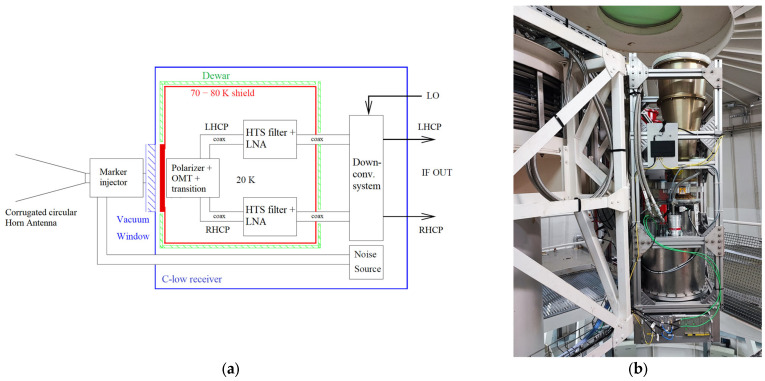
(**a**) Block diagram of the dual-polarized C-low receiver infrastructure; (**b**) Photo of the dual-polarized cryogenic C-low receiver installed on the Gregorian receiver positioner of the Sardinia Radio Telescope (SRT).

A block diagram of the entire dual-polarized C-low receiver infrastructure and a photo of the system installed on the Gregorian receiver positioner of SRT are shown in Figure 1a and Figure 1b, respectively.

The main components of the dual-polarized cryogenic C-low receiver infrastructure are the following:

- The feed system, which is composed of a horn antenna, a marker injector, a vacuum window, a polarizer and an ortho-mode transducer (OMT), permits the detection of radio frequency signals from the sky. In particular, a corrugated circular horn antenna [37] is directly connected to a microwave directional coupler to inject a signal used to calibrate the receiver (marker injector) [38]. Downstream of this, there is a vacuum window that allows entry into the cryostat (i.e., the Dewar), where the rest of the feed system is housed. This includes the so-called polarizer, which is essentially a differential phase shifter (DPS) that enables the conversion of the detected electromagnetic radiation’s polarization from linear to circular [39]. Finally, the OMT is responsible for separating the two polarizations: right-hand and left-hand circular polarizations (RHCP and LHCP), respectively [39]. For each OMT output, there is a waveguide-to-coaxial transition that allows the conversion from the waveguide domain to the coaxial domain [40].
- The Dewar (cryostat) represents a mechanical structure that enables the internal environment to reach cryogenic temperatures below 20 K, helping the system achieve low noise temperatures [41]. It is connected to a system that enables vacuum generation and to a CTI-Cryodyne cold head, model 350 CP, which allows reaching the desired cryogenic temperature [42]. In addition to the polarizer, the OMT and the waveguide-to-coaxial transition, the Dewar houses a High Temperature Superconductor (HTS) filter and the cryogenic low noise amplifier (LNA) for each polarization channel. The HTS filter consists of a cascaded band-pass stage with a bandwidth matching the receiver’s operating band (i.e., 4.2–5.6 GHz) and a notch filter that rejects a strong interference at 5640 MHz present at the SRT site, emitted by a weather radar installed in the surrounding area (see Section 2.2 for further details on the RFI scenario around SRT in the frequency range of interest) [43]. The primary purpose of the filter is to constrain the operating bandwidth to the receiver’s specifications, attenuating out-of-band signals. The LNA is the LNF-LNC4_8C model from Low Noise Factory (LNF) [44], which, at a physical temperature of 5 K, provides a gain of approximately 42 dB and a noise temperature below 2 K within the 4.2–5.6 GHz frequency range. The electromagnetic characterization of these components is detailed in [28].
- The noise source block permits the calibration of the system. A coaxial noise generator (model ATM NX3248Y from Narda/Miteq [45]) with an excess noise ratio (ENR) of approximately 31 dB at 5 GHz, is connected to a coaxial attenuator of 24 dB (model R4118xx1yy from Radial [46]). It is directly connected to the marker injector described above via waveguide-to-coaxial transition. Thanks to this calibration block, accurate system temperature (Tsys) measurements can be performed with the telescope, and the corresponding values will be reported for each characterization test. The Tsys combines the antenna temperature (TA), which includes contributions from sky, ground, atmospheric emission, and possible RFI, with the receiver noise (Trec) [47]. Variations of a few kelvins, up to approximately 5–10 K, are therefore expected due to changes in atmospheric conditions, elevation-dependent ground spillover, residual calibration uncertainties, and presence of strong RFI, and do not indicate any degradation of the receiver performance.
- The down-conversion system shifts the radio frequency band of 4.2–5.6 GHz to the SRT baseband of 0.1–1.5 GHz. It consists of two cascade down-conversion stages in order to minimize intermodulation products. The first stage down-converts the radio frequency (RF) band from 4.2–5.6 GHz to 2.6–4.0 GHz, while the second one generates the final intermediate frequency (IF) range of 0.1–1.5 GHz. The entire block has a gain of approximately 45 dB and noise figure of about 6 dB at the center of the IF band [28]. A detailed description of all components of the down-conversion system is provided in [28].
- The monitoring and control unit permits the biasing of the LNAs, the remote control and monitoring of the vacuum system and the cold head [28].

**Figure 2:**
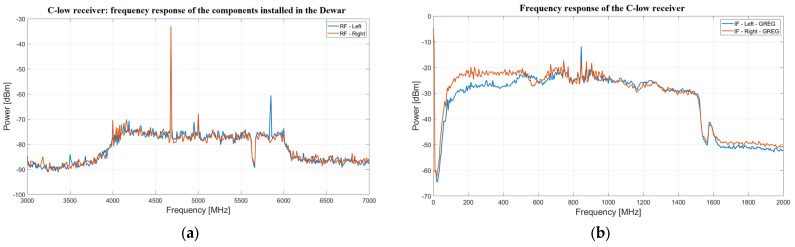
(**a**) Frequency response of the radio frequency chain inside the Dewar. RF-left and RF-right refer to the LHCP and RHCP channels, respectively; (**b**) Frequency response of the entire C-low receiver, which has an intermediate frequency between 0.1 and 1.5 GHz that coincides with the SRT baseband. IF-left-GREG and IF-right-GREG refer to the intermediate frequency (IF) signals measured in the Gregorian room of the SRT for the LHCP and RHCP channels, respectively.

Figure 2a shows the frequency response of the components installed inside the Dewar for both LHCP and RHCP polarization channels, with the receiver positioned out of focus inside the Gregorian room. This out-of-focus simulation replicates the effect of placing a warm calibration load at the receiver feed aperture, as used to characterize the system response. The measurement was performed using a spectrum analyzer, with a frequency window ranging from 3 to 7 GHz (i.e., the span parameter of the spectrum analyzer) and a resolution bandwidth (RBW) of 100 kHz. Several RFIs can be observed, along with a band notch at 5640 MHz due to the aforementioned HTS filter [43]. It can also be observed that the frequency response of the Dewar components, excluding the down-conversion stage, is broader (i.e., 4.2–6 GHz) than the target bandwidth of 4.2–5.6 GHz selected for the C-low receiver. This is because the chosen cryogenic LNAs operate over a wider frequency range.

Instead, Figure 2b shows the frequency response of the entire C-low receiver for both LHCP and RHCP polarization channels. As clearly shown in the figure, the receiver’s IF ranges from 0.1 GHz to 1.5 GHz, which coincides with the SRT baseband. Unfortunately, the LHCP channel exhibits a signal level approximately 6 dB lower than that of the RHCP channel in the first 500 MHz of bandwidth. This critical issue is related to the down-conversion system technology, and in the future, an upgrade could be considered to implement a new system that ensures equalized LHCP and RHCP channels.

**Figure 3:**
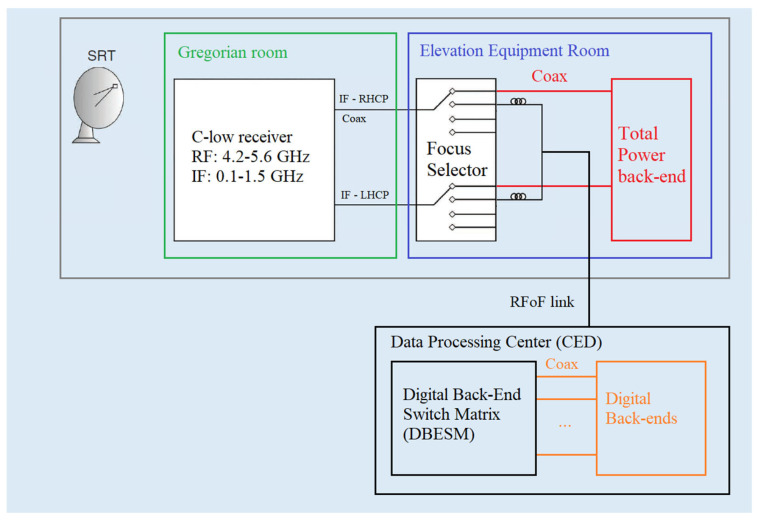
Block diagram of the entire signal acquisition chain of the Sardinia Radio Telescope.

To store and process the data acquired by the receiver, it must be connected to the digital back-end. Specifically, the receiver’s IF outputs must be integrated into the entire SRT signal acquisition chain (see Figure 3).

In particular, the IF outputs of the receiver are directly connected to the so-called focus selector, which consists of a set of amplifiers, variable attenuators, switches and radio frequency filters, installed on the Elevation Equipment Room (EER) of SRT [22,23]. This system is used to select the IF signals from the different receivers installed at the different focal points of SRT during observations. After passing through the focus selector, the signal is either transmitted via a Radio Frequency-over-Fiber (RFoF) link, using fiber optics, to the data processing center (CED) located 500 m away from the SRT, or to the TP back-end, which is installed directly in the EER area. The CED is housed inside a shielded room to minimize the RFI generated by the electronic systems it contains, such as the digital back-ends available for SRT. For the distribution of the IF signals, which are converted back from optical to radio frequency signals, to the digital back-ends (i.e., DFB3, DBBC and SARDARA), the CED includes a board called the Digital Back-End Switch Matrix (DBESM) [28]. This board also conditions the signal through additional amplification stages and variable attenuators. The DBESM is controlled by proprietary software, allowing users to optimize the amplitude levels of the receiver channels used during observations. In the case of the C-low receiver, it is possible to adjust the attenuation levels of the lower part of the band (see Figure 2b) to equalize the two polarization channels.

### 2.2. Scenario of Radio Frequency Interference Detectable by the Sardinia Radio Telescope with Its C-Low Receiver

As mentioned in the Introduction, the 4.2–5.6 GHz frequency window is affected by various sources of RFI, including emissions from radar, terrestrial and satellite communications, and wireless transmissions. Understanding these sources and evaluating their frequency occupancy is crucial to assessing their impact on scientific observations.

**Figure 4:**
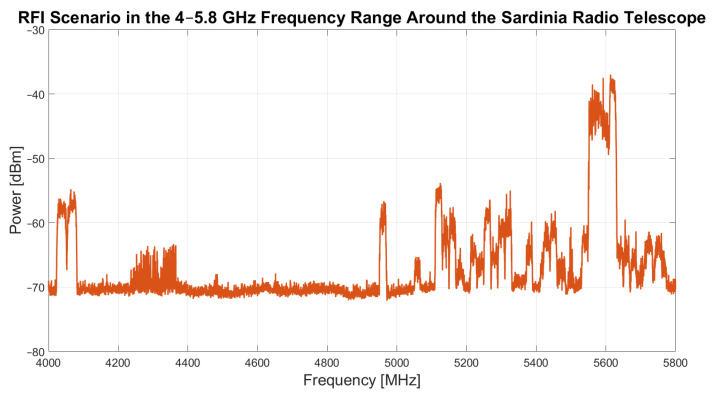
Radio frequency interference in the 4–5.8 GHz frequency range around the Sardinia Radio Telescope (SRT) [18].

Figure 4 presents the results of an RFI measurement campaign conducted in October–November 2023 in the 4–5.8 GHz frequency range, highlighting the presence of RFI from the area surrounding the SRT. This spectrum provides valuable insights for astronomers using the SRT for scientific research, helping to develop mitigation strategies and preserve the radio astronomical environment for future observational campaigns. Further details about the measurement setup, type of detected signals and directions of the source are discussed in [18]. In addition to signals from the surrounding area, special attention must be given to potential self-generated signals from the electronic instrumentation installed on the antenna. For this reason, periodic monitoring and updates of RFI maps are essential to facilitate the smooth execution of scientific observations.

## 3. Results of the Characterization of the C-Low Receiver and Technical Discussion

Characterization tests—including measurements of pointing accuracy, focus, gain curves, and beam shape—were carried out on the SRT C-low receiver between May 2024 and January 2025 to evaluate the antenna’s performance and to optimize its observational capabilities across the receiver’s operating frequency band. Performing such measurements is particularly crucial after major modifications or maintenance operations on the antenna, as they ensure the proper functioning of key subsystems such as the active surface, servo systems, receivers, digital back-ends and the software control system.

The same experimental setup was employed for all tests. Specifically, the shaped mode of the active surface was used, and the SRT operated in tracking mode. The TP back-end was utilized, selecting the focus selector’s band-pass filter with a 300 MHz bandwidth. The local oscillator of the C-low receiver was tuned to 4.6 GHz in order to consider the frequency band 4.6–4.9 GHz. This frequency range was selected because, as shown in Figure 4, it is free from RFI, allowing the use of the TP back-end instead of SARDARA, which would have been necessary in the presence of RFI within the observing band. Both the sampling interval and the integration time were set to 40 milliseconds. Before each experiment, the Tsys was measured, and the meteorological conditions were recorded.

The results of each characterization test are presented in the following Sections: Section 3.1 describes the pointing model, Section 3.2 the focus curve calibration, Section 3.3 the gain curve calibration, and Section 3.4 the beam shape analysis.

### 3.1. Pointing Accuracy Model

The pointing model is essential for verifying and enhancing the antenna’s pointing accuracy. The strategy involves performing on-the-fly (OTF) cross-scans in the horizontal frame on a large sample of pointing calibrators [28], selected to ensure uniform sky coverage in both azimuth and elevation over the entire sky (generally requiring observation sessions of at least six hours). These observations are typically carried out at night, avoiding sunrise and sunset, to minimize thermal effects on the antenna structure [48,49]. For each cross-scan the pointing offset with respect to the source direction is measured and, once the azimuth/elevation plane has been adequately sampled, the data are fitted using a polynomial pointing model [50] through pdplt, a tool within the VLBI Field System [51,52,53]. The updated pointing parameters are then implemented in DISCOS [28] and validated in a subsequent test session. The model is considered satisfactory when the residuals in both azimuth and elevation (i.e., the pointing offsets relative to the source direction) are less than one-tenth of the beam size.

The pointing model for the C-low receiver was performed on 21 May 2024. The observation campaign was carried out using cross-scans of bright quasars, spanning the entire elevation range from 10 degrees to 80 degrees. Each cross-scan was executed over a length of 0.7 degrees (42 arcminutes), with the antenna moving at a constant speed of 3 arcminutes per second. Before the start of the experiment, Tsys values of 28 K and 31 K were recorded for the two polarization channels, respectively. Meteorological conditions were also documented at the time of observation, with an ambient temperature of about 18 °C, relative humidity of 66%, atmospheric pressure of 945 hPa, wind speed of about 8 km/h, and wind direction of 249° from North. The results of this characterization are reported in Table 1.

The model is absolutely satisfactory because the root-mean-squared (RMS) of pointing offsets relative to the source direction (i.e., azimuth RMS and elevation RMS equal to 0.00319 degrees and 0.00273 degrees, respectively) is at most 0.0032 degrees (this value is named blind pointing accuracy), which is less than one-tenth of the theoretical beam size (which corresponds to a loss of gain of 0.13 dB for pointing errors) at the observed frequency, which is approximately 0.065 degrees.

### 3.2. Focus Curve Calibration

Maintaining the correct focus of the telescope is essential, as temperature gradients and other atmospheric effects can compromise its stability, directly impacting data quality. As previously noted, SRT is a multi-reflector system. In particular, it is equipped with a 7.9-m sub-reflector, referred to as M2, which has six degrees of freedom—three translational and three rotational motions along the x, y, and z axes (which we may label as a, b, c, d, e, and f). Among these axes, the most critical is the z-axis, since displacements along the x and y axes can be compensated through antenna pointing adjustments. Dedicated measurements are therefore required to monitor the focal shift as a function of the antenna’s elevation. It is important to note that, in order to accurately determine the optimal focal position, the scan length of M2 should be at least three times the observing wavelength (the wavelength is approximately 190 mm for the C-low experiments). The obtained focus curve (focus offset vs. elevation pointing) is fitted with the following 5th-degree polynomial [28]:

(1) $$y=a\cdot x^{5}+b\cdot x^{4}+c\cdot x^{3}+d\cdot x^{2}+e\cdot x+f\;$$

where each coefficient (*a*, *b*, *c*, *d*, *e*, and *f*) is determined.

The focus measurements of the SRT with the C-low receiver were carried out on 17 July 2024. The calibrators used for focus adjustments must share the same characteristics as those employed for pointing measurements (see Section 3.1). Before starting the observation, the Tsys of SRT was measured for both polarization channels, yielding values of 22.7 K and 24.6 K, respectively. Environmental conditions were also logged at the time of the measurements: the ambient temperature was approximately 30 °C, with a relative humidity of 46.1%, atmospheric pressure of 949 hPa, wind blowing at about 7 km/h, and a wind direction of 94° from North.

**Figure 5:**
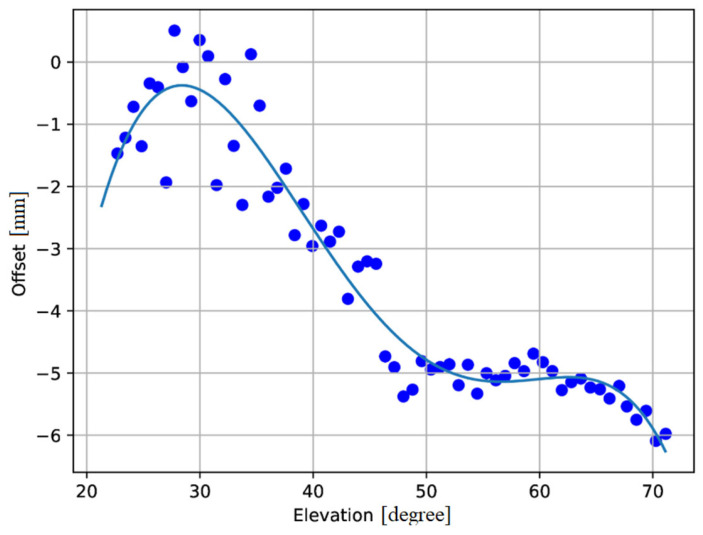
Offset in millimeters of the focal position (z-axis) as a function of the Sardinia Radio Telescope (SRT) elevation. The blue points represent the measurements, while the focus curve is obtained by evaluating the 5th-degree polynomial in Equation (1).

Figure 5 shows the focus curve, where the focal position in millimeters (along the z-axis) is plotted as a function of the antenna elevation. The blue points represent the measurements, while the curve is obtained by evaluating the polynomial in Equation (1). The observed trend describes the effects induced by the gravitational load acting on the sub-reflector M2 and on the quadruped structure that supports it. The corresponding coefficients of the 5th-degree polynomial in Equation (1) (i.e., *a*, *b*, *c*, *d*, *e*, and *f*) are reported in Table 2. An analysis of the coefficients of the 5th-degree polynomial used to model the focus curve reveals that the coefficients *e* and *f* exhibit a high degree of uncertainty. This indicates greater variability in their estimates, suggesting that their contribution to the model may not be statistically significant.

### 3.3. Gain Curve Calibration

Determining the antenna gain curve consists in measuring and modeling how the antenna gain varies with elevation. Since the structural deformation of the antenna, atmospheric attenuation, and alignment errors can affect performance at different elevation angles, this calibration is essential for correcting observational data. The result is a gain curve, a plot of the antenna gain as a function of elevation, which is used to ensure accurate flux density measurements and optimize the telescope’s sensitivity across the sky. Gain is commonly defined as degree per flux unit, and it is expressed in K/Jy.

One can fit a 2nd-degree polynomial to the measured gain curve (i.e., gain vs. elevation):

(2) $$Gain\;\left[\frac{K}{Jy}\right]=C_{0}\cdot El^{2}+C_{1}\cdot El+C_{0}$$

with the parameters *C*_0_, *C*_1_ and *C*_2_.

The gain curve measurements of the SRT with the C-low receiver were carried out on 19 July 2024.

The observing strategy consisted of cross-scans of 3C84 covering the full elevation range from 10 degrees to 80 degrees, with additional cross-scans of 3C147 at an elevation of approximately 50 degrees to obtain the conversion from K to Jy and derive the gain curve in K/Jy. Each scan covered an area of 30 arcminutes × 30 arcminutes and was performed with an antenna scanning speed of 3 arcminutes per second.

The observing schedule for this characterization test included pointing and focus operations at the beginning of the session and during either sunset or sunrise, using bright radio calibrators such as 3C84, 3C295, 3C123, and 3C286.

Prior to the experiment, Tsys values of 29.5 K and 32.3 K were measured for the two polarization channels, respectively. Additionally, weather data were recorded, including a temperature of approximately 30 °C, humidity of 38.3%, atmospheric pressure of 950.3 hPa, wind speed of about 12 km/h, and wind direction of 104° from North.

Data reduction was performed using the proprietary Single-dish Spectral-polarimetry Software (SCUBE) [54]. The flux density of the bright radio calibrator 3C84 was obtained by calibrating it against 3C147, observed at the same elevation, which has a known flux of 7.88273 Jy. The resulting calibrated flux of 3C84 was found to be 44.1751 Jy.

**Figure 6:**
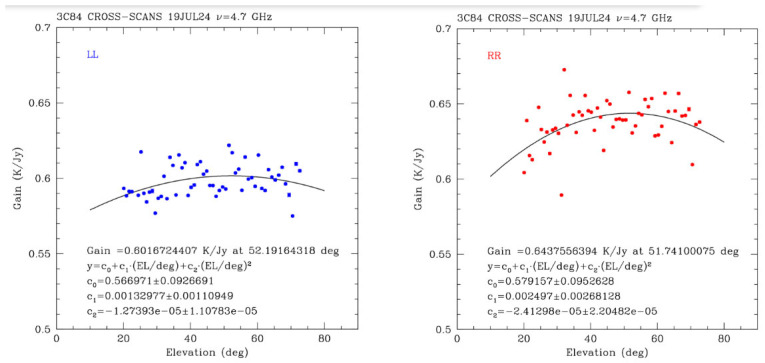
Results of the gain curve calibration for the both polarization channels (LL in blue indicates LHCP, and RR in red indicates RHCP) of the C-low receiver of SRT.

**Figure 7:**
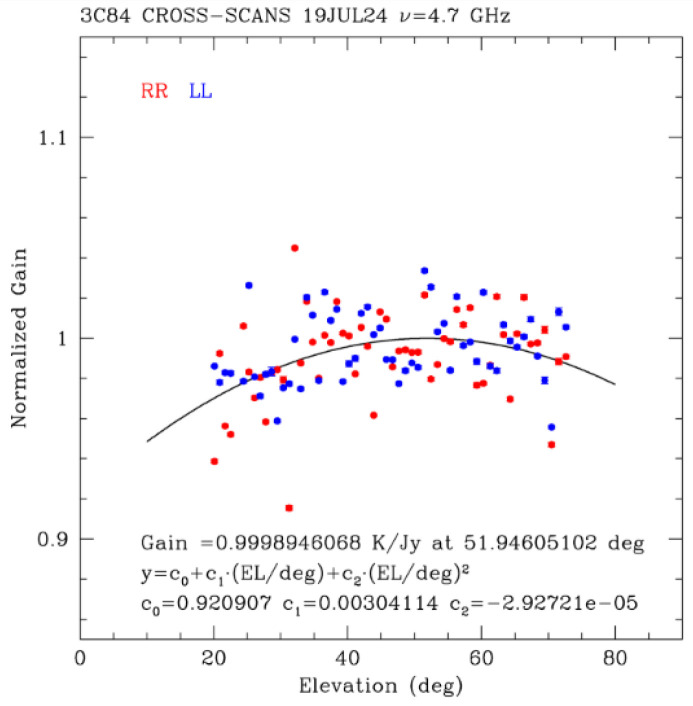
The overall normalized gain curve for both polarization channels (LL in blue indicates LHCP, and RR in red indicates RHCP) of the C-low receiver of SRT.

The results of the measurements fitted with the 2nd-degree polynomial in Equation (2) are plotted in Figure 6, obtaining a peak gain of 0.60 K/Jy at 52.2 degrees of elevation (LHCP channel) and a peak gain of 0.64 K/Jy at 51.7 degrees of elevation (RHCP channel). An overall normalized gain curve is also reported in Figure 7.

The ideal gain curve should be flat to guarantee a constant gain at all elevations. The gain curve obtained with the C-low receiver (see Figure 6 and Figure 7) is already very good, but it could still be further improved in the near future through better alignment of the main 64-m reflector M1 and the 7.9-m sub-reflector M2 of SRT, enabled by ongoing metrology activities [55].

In Table 3, a summary of the coefficients C_0_, C_1_ and C_2_ of the 2nd-degree polynomial (2), referring to a peak gain of 0.60 K/Jy at 52.2 degrees of elevation (LHCP channel) and to a peak gain of 0.64 K/Jy at 51.7 degrees of elevation (RHCP channel), is listed.

### 3.4. Beam Shape Characterization

The radiation pattern of SRT using its C-low receiver, which describes the angular distribution of the received power as a function of direction, is commonly referred to as the beam shape in radio astronomy, where it represents the telescope’s response across the sky. Therefore, beam shape characterization in total intensity is a key tool for evaluating potential variations or aberrations as a function of elevation and for verifying the accuracy of the pointing model of SRT associated with the C-low receiver. In addition, the evaluation of the beam size gives reliable information on the optics of the telescope and on the good functioning of the active surface and tracking sub-reflector M2.

The resulting test, carried out on 15 January 2025, consists in evaluating the percentage contribution of the second and third lobes with respect to the central beam. Observations were performed using cross-scans on the astronomical radio source 3C84 covering elevations from 23 degrees to 80 degrees. Each scan extended 0.7 degrees (42 arcminutes) and was performed at a constant rate of 4 arcminutes per second. The measured Tsys was 29.6 K for the LHCP channel and 28.3 K for the RHCP channel. Meteorological measurements recorded a temperature of about 8 °C, relative humidity of 63.5%, atmospheric pressure of 948.5 hPa, wind speed of about 4 km/h, and wind direction of 311° from North.

**Figure 8:**
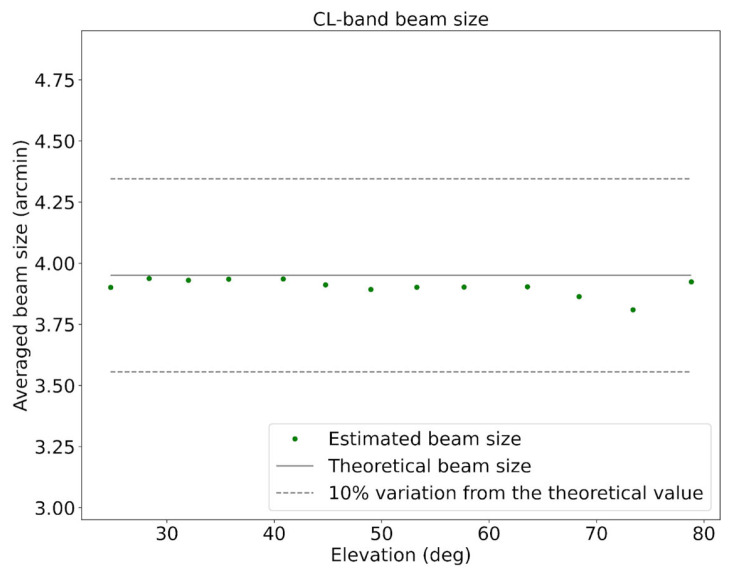
Variation of the beam size as a function of elevation, obtained from the beam shape characterization conducted on 15 January 2025.

The results of the beam shape characterization show a beam size that remains stable across elevations, with an average value of approximately 3.9 arcminutes. The variation of the beam size as a function of elevation is shown in Figure 8.

**Figure 9:**
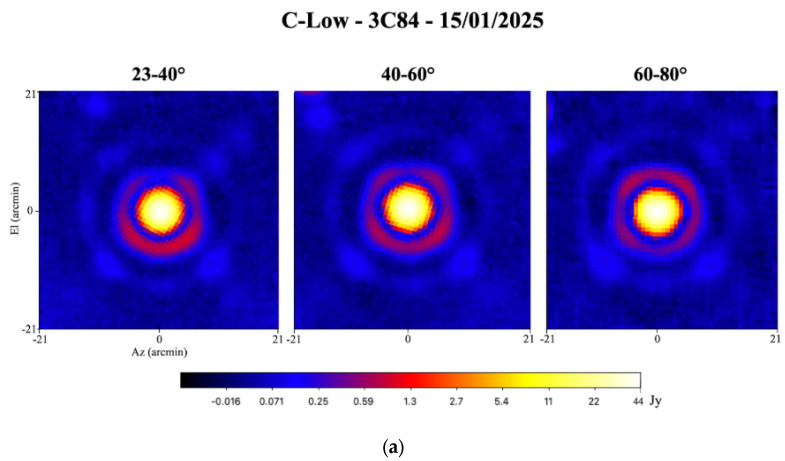

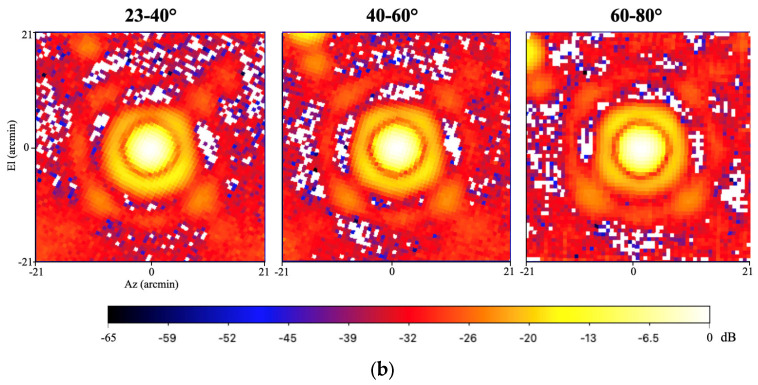
(**a**) Az/El maps of 3C84 obtained at 4.8 GHz with the Total Power backend on 15 January 2025, expressed in Jy. (**b**) Az/El maps of 3C84 obtained at 4.8 GHz with the Total Power backend on 15 January 2025, ex-pressed in dB.

The resulting maps of the beam shape characterization, obtained using the proprietary Single Dish Imager (SDI) software [56], are shown in Figure 9a in Jy. Representing the beam in terms of gain (dB) versus angle (see Figure 9b), as in a conventional antenna pattern, highlights the contributions of the struts and other structural features, making them more clearly interpretable for antenna engineers. The second lobes of the radiation pattern and the SRT quadruped (support structure that holds the sub-reflector M2 of the SRT) are visible on the maps. The second lobe is slightly asymmetric at low and high elevations (i.e., 23–40° and 60–80°), as already noticed for the C-high and K-band receivers at SRT [28]. Instead, it appears more symmetric at 40–60° of elevation.

Finally, the flux density values of 3C84—corresponding to the central lobe and the secondary lobes (i.e., the brightest pixels in the second and third lobes) for the various elevation ranges—are listed in Table 4.

## 4. Conclusions and Future Work

This article has presented the C-low receiver of the SRT, detailing its design, signal acquisition chain, and the results of characterization tests on pointing accuracy, focus and gain calibrations, and beam-shape analysis. These tests demonstrate that the receiver achieves stable and precise performance, meeting stringent operational requirements.

By extending SRT’s capabilities in the lower C-band, the C-low receiver enables high-sensitivity observations of a wide range of astrophysical sources, including pulsars and transient events, making it a robust and versatile instrument for both current and future scientific programs.

Showing very good performance, the C-low receiver has been offered to the scientific community since 2024. It is used for both single-dish and VLBI projects, as it is part of the European VLBI Network [33]. Notably, it has been employed to monitor micro-quasars [57] and active galactic nuclei (AGNs) during flaring activities.

In parallel, thermal effects on the SRT structure have been analyzed through finite element analyses, showing solar-induced pointing offsets of several tens of arcseconds, significantly smaller than gravitational deformations [48]. Owing to the thermal inertia of the steel structure, these effects can be considered quasi-stationary over time scales of about ten minutes and can therefore be estimated and corrected. While investigations of thermal effects on the primary mirror surface accuracy are ongoing, dedicated studies on thermal-induced focus variations are still missing. However, thermal loads acting on the quadruped structure affect the sub-reflector position, impacting both focus and beam shape, and will be addressed in future work.

## Figures and Tables

**Table 1:** Pointing model for the C-low receiver of SRT.

Parameter	Explanation	Value [Degree]
P_1_	Azimuth encoder offset	−2.2894389629
P_2_	Gravity effect on the RF axis projected on azimuth	0.0000000000
P_3_	Axis skew	0.0002044142
P_4_	Box Offset	0.0018049411
P_5_	Tilt out (tilt of El = 90° toward az/el = 0°,0°)	−0.0000764903
P_6_	Tilt over(tilt of El = 90° toward az/el = 90°,0°)	−0.0015140247
P_7_	Elevation encoder offset	0.0495181828
P_8_	Gravity effect on the RF axis projected on elevation	0.1174282282
P_9_	Elevation angle slope	0.0000000000
P_10_	ElcosEl	0.0000000000
P_11_	ElsinEl	0.0047037964
P_12_	Azimuth angle slope	0.0000000000
P_13_	AzcosAz	0.0004461684
P_14_	AzsinAz	−0.0008204504
P_15_	Elcos2Az	0.0000000000

**Table 2:** Values of all the coefficients of the 5th-degree polynomial used to fit the data and derive the focus curve.

Parameter	Value [mm]
a	−4.14149825680894 × 10^−08^ ± 3.9266120827404615 × 10^−14^
b	−6.156655562744601 × 10^−06^ ± 2.160409681147414 × 10^−09^
c	0.0022361557302186348 ± 1.7982894322074104 × 10^−05^
d	−0.18064945141023792 ± 0.03520549739754111
e	5.548988532710736 ± 16.133021451316196
f	−58.71312982483134 ± 1103.239053358789

**Table 3:** List of the coefficients C_0_, C_1_ and C_2_ referring to a peak gain of 0.60 K/Jy at 52.2 degrees of elevation (LHCP channel) and to a peak gain of 0.64 K/Jy at 51.7 degrees of elevation (RHCP channel).

Parameter	4.7 GHz [K/Jy]—LHCP	4.7 GHz [K/Jy]—RHCP	4.7 GHz—Normalized
C_0_	0.57 ± 0.09	0.58 ± 0.1	0.920907
C_1_	0.0013 ± 0.0011	0.0025 ± 0.0027	0.00304114
C_2_	−1.3 × 10^−5^ ± 1.1 × 10^−5^	−2.4 × 10^−5^ ± 2.2 × 10^−5^	−2.92721 × 10^−5^

**Table 4:** Flux density of 3C84 associated with the central beam and the secondary lobes (brightest pixel in the second and third lobes) for the different elevation pointing ranges obtained at 4.8 GHz on 15 January 2025. The percentages of the second and third lobes correspond to the contribution of the counts in each lobe relative to the central one.

3C84 at 4.8 GHz	El Pointing of 23–40° [Jy]	El Pointing of 40–60° [Jy]	El Pointing of 60–80° [Jy]
Flux density of 3C84 (central lobe in Jy)	43.4	42.3	42.4
Second lobe (brightest pixel in Jy)	0.96 (2.2%)	0.78 (1.8%)	0.73 (1.7%)
Third lobe (brightest pixel in Jy)	0.18 (0.46%)	0.20 (0.47%)	0.19 (0.45%)

## Data Availability

The original contributions presented in this study are included in the article. Further inquiries can be directed to the corresponding authors.
